# Supplementary material for: Seminoma and Embryonal Carcinoma Footprints Identified by Analysis of Integrated Genome-Wide Epigenetic and Expression Profiles of Germ Cell Cancer Cell Lines
Source: PLoS One. 2014 Jun 2;9(6):e98330. doi: 10.1371/journal.pone.0098330 (PMC4041891; doi:10.1371/journal.pone.0098330)
Supplement: File S1 — ZIP file containing DMRforPairs output for significant regions. Please start from the html files. (ZIP) [file pone.0098330.s008.zip › figures/19810.pdf]

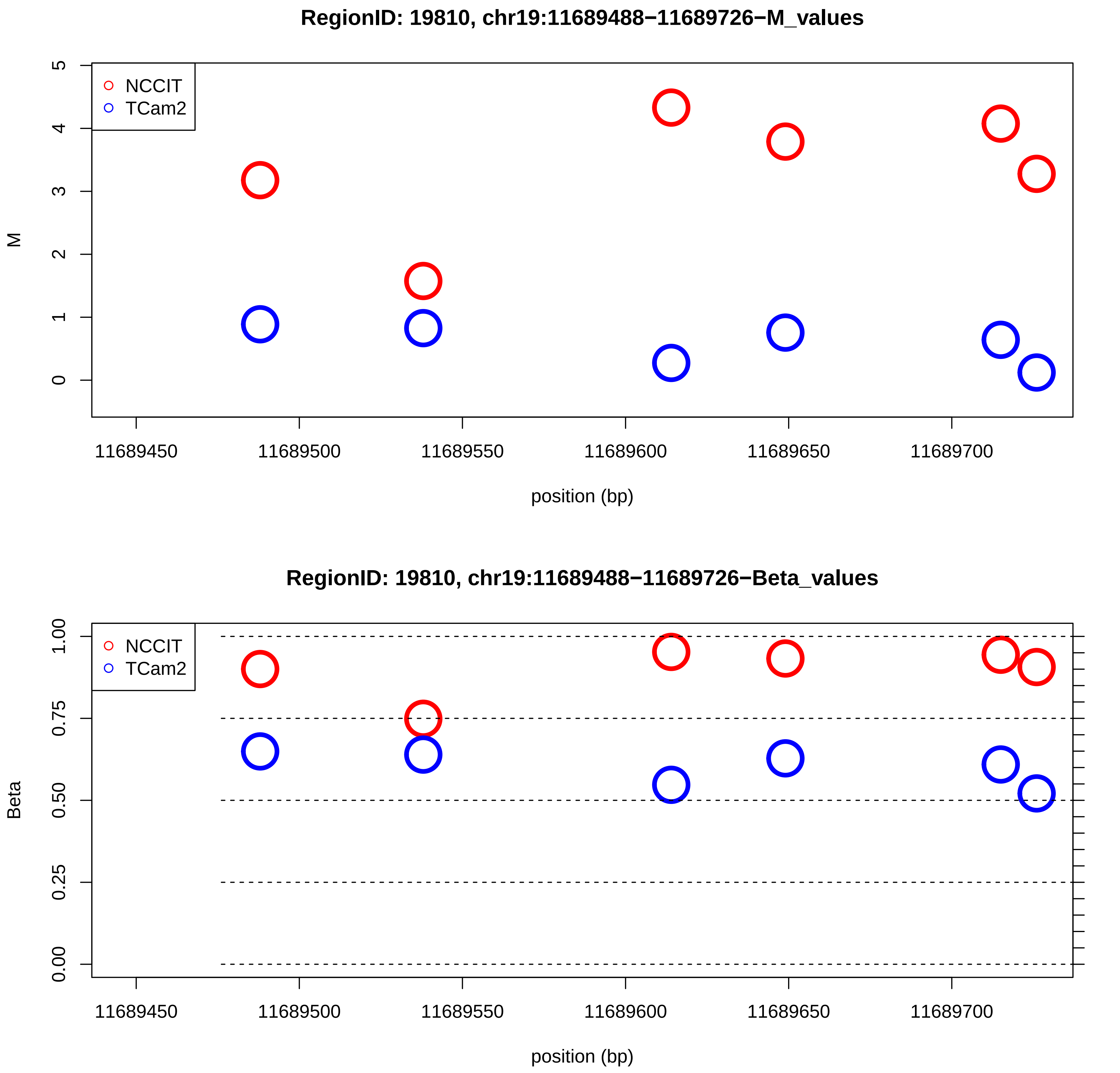
RegionID: 19810, chr19:11689488−11689726−M_values
NCCIT
TCam2
M
0
1
2
3
4
5
11689450
11689500
11689550
11689600
11689650
11689700
position (bp)
RegionID: 19810, chr19:11689488−11689726−Beta_values
NCCIT
TCam2
Beta
0.00
0.25
0.50
0.75
1.00
11689450
11689500
11689550
11689600
11689650
11689700
position (bp)
